# Effort-dependent effects on uniform and diverse muscle activity features in skilled pitching

**DOI:** 10.1038/s41598-021-87614-z

**Published:** 2021-04-15

**Authors:** Tsubasa Hashimoto, Ken Takiyama, Takeshi Miki, Hirofumi Kobayashi, Daiki Nasu, Tetsuya Ijiri, Masumi Kuwata, Makio Kashino, Kimitaka Nakazawa

**Affiliations:** 1 Department of Electrical Engineering and Computer Science, Tokyo University of Agriculture and Technology, 2-24-16, Nakacho, Koganei, Tokyo Japan; 2 Department of Life Sciences, Graduate School of Arts and Sciences, The University of Tokyo, Tokyo, Japan; 3 NTT Communication Science Laboratories, Nippon Telegraph and Telephone Corporation, Atsugi, Kanagawa Japan

**Keywords:** Motor control, Human behaviour

## Abstract

How do skilled players change their motion patterns depending on motion effort? Pitchers commonly accelerate wrist and elbow joint rotations via proximal joint motions. Contrastingly, they show individually different pitching motions, such as in wind-up or follow-through. Despite the generality of the uniform and diverse features, effort-dependent effects on these features are unclear. Here, we reveal the effort dependence based on muscle activity data in natural three-dimensional pitching performed by skilled players. We extract motor modules and their effort dependence from the muscle activity data via tensor decomposition. Then, we reveal the unknown relations among motor modules, common features, unique features, and effort dependence. The current study clarifies that common features are obvious in distinguishing between low and high effort and that unique features are evident in differentiating high and highest efforts.

## Introduction

High-speed throwing has been a fundamental motion for *Homo sapiens* in the past two hundred years^1^—for instance, it was a motion used for animal hunting a long time ago and is still used for baseball, football, handball, track and field, and a wide variety of sports. Fastballs thrown by baseball pitchers are one of the most representative and fastest examples of high-speed throwing.

Even today, the fastball pitch is still evolving, especially in baseball. An article in *The Washington Post* mentioned the increase of the fastball velocity in major league baseball (MLB) in the past ten years, and faster fastballs have resulted in lower hitting rates by opposing batters^2^. Fastball velocity can thus be a key factor for a sophisticated performance in baseball. In contrast, a faster fastball substantially increases the possibility of injury to the pitcher’s elbow^2, 3^. Elucidating the motion features associated with a high throwing speed can provide insight that can be used for improving sports performance and lowering the risk of injury.

The motion features that contribute to higher and lower pitching speeds have been investigated in both between-subject and within-subject manners. In a between-subject manner, faster baseball pitchers showed different kinematic and temporal factors relative to slower pitchers^4^. Kinetic parameters, rather than joint positions and temporal parameters, showed differences among youth, high school, college, and professional pitchers^5^. In a within-subject manner, by dividing the throwing motions of the maximum effort by each pitcher into faster ball-velocity trials and slower ball-velocity trials, the kinematic, kinetic, and temporal parameters associated with faster balls were investigated^6, 7^.

The within-subject differences were examined not only in maximum effort pitches but also in three levels of effort or pitching velocity. A previous study investigated throwing motions while the subject was seated (referred to as unskilled throws in an earlier study^8^) with slow, medium, and maximum speeds^9^. The study revealed that muscle torques in proximal joints (e.g., shoulder) produced interaction torques in more distal joints (e.g., elbow and wrist). The interaction torque in the elbow via more proximal joints accelerated elbow angular velocity, which was key to achieving high ball speed. Similar relations were also observable among slow, medium, and maximum speeds in skilled pitching (i.e., usual throwing motions from wind-up to cocking, release, and follow-through while standing)^10^. Pitchers thus commonly accelerate wrist and elbow joint rotations via proximal joint motions across the three levels of pitching velocity.

In contrast to the common motion features, each pitcher throws a fastball with a diverse form setup with differences evident in their stride, arm-cocking, release, and follow-through. Pitching motion thus consists of common and unique motion features. The unique motion features originate from the redundancy inherent in our motor system. Due to redundant numbers of joints and muscles, the same motion result (e.g., pitching a ball toward the same location) is achievable via diverse motion repertoires. It is thus reasonable that pitching velocity differs among individuals under the same ball endpoint. Because the difference in pitching velocity affects pitching performance and injury risk^2, 3^, it is indispensable to study diverse motion features. The diverse motion features are compatible with the uniform motion features in pitching motions, roll-and-rise motions^11^, drum hitting^12^, running^13^, piano playing^14^, and diverse motion repertoires.

Despite the universality of common and unique motion features, it remains unclear how motion effort modulates these features. In other words, the relation of a faster fastball to common and diverse motion features is unknown. In playing the piano, pianists can be classified into clusters depending on effort-dependent effects on kinematic parameters^14^. Similarly, a possibility is that unique motion features can be more evident when greater effort is exerted in skilled pitching. Another possibility is that greater effort is associated with more evident common motion features because common features play roles in facilitating faster elbow and wrist rotations^10^. Notably, there are other possibilities for how uniform and diverse motion features depend on motion effort.

Here, we investigate effort-dependent effects on common and diverse motion features in skilled pitching. Specifically, we examine electromyographic (EMG) activity in skilled throws with three levels of effort or ball speeds. In contrast to kinematics and kinetics, fewer studies have measured muscle activity in pitching motions. In unskilled throws with slow, medium, and maximum speeds, the wrist EMG activity was not directly associated with the muscle torque around release timings at the wrist joint, indicating the relation of the interaction torque to the muscle torque^8^. In skilled throws with maximum effort, muscle activities showed sequential timings of peak activities^15^. Although EMG activities in unskilled throws with three levels of effort^8^ and skilled throws with maximum effort have been discussed^15^, few studies have investigated EMG activities in skilled throws with different levels of effort or ball speeds. Elucidating effort-dependent effects on EMG features can provide insight into ways to control the fastball velocity, improve the pitching performance, and decrease the risk of injury.

In particular, the current study reveals the features of EMG activities relevant to motion effort from the perspective of muscle synergy or a muscle module^16–18^. In the module hypothesis, the central nervous system (CNS) controls muscle activity while grouping muscles rather than evaluating muscles independently. The number of muscles, or the number of degrees of freedom (DoF), is tremendous and more than what is necessary to achieve desired movements in massive cases. For example, let us consider the case of throwing a ball at a (not so fast) release speed. Although it is possible to achieve the motion by rotating only an elbow while recruiting at least two related muscles, we sometimes realize the motion by moving the elbows, shoulders, wrists, trunk, and legs while recruiting dozens or hundreds of muscles. The latter case denotes an example of the redundant number of DoFs required to achieve the desired movement. A possible way to reduce the redundant number of DoFs is to group muscles that are needed to achieve desired movements. The groups of muscles and these recruitment patterns are hereafter referred to as spatial modules and temporal modules, respectively. The spatial and temporal modules can originate from spinal cord activities^16, 17^ and provide insight into how muscle activities are related to motor repertoires, such as locomotion^18^, arm-reaching movements^19^, and maintaining balance while standing^20^.

**Figure 1:**
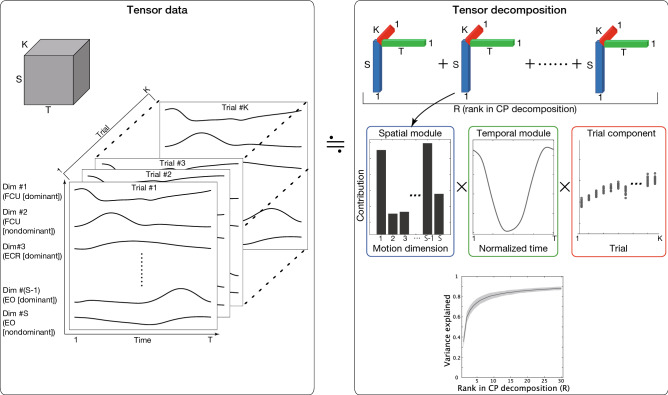
Summary of CP decomposition, a variant of tensor decomposition. (Left): Tensor data consist of three factors: S columns, T rows, and K slices of matrices. In the current study, S, T, and K denote the number of muscles, the number of time frames, and the number of trials, respectively. We defined one tensor data point in each subject with all the trials and effort (i.e., the number of trials was 30 including 10 trials for 50% effort, 10 trials for 80% effort and 10 trials for 100% effort). (Upper-right and middle-right): CP decomposition provides us with R rank-1 tensors, referred to as tensors for simplicity throughout this study. Each tensor includes a spatial module (the bar graph surrounded by a blue rectangle in the middle panel), a temporal module (the line plot surrounded by a green rectangle in the middle panel), and a trial component to indicate the trial-dependent or effort-dependent effects on the spatiotemporal module (the scatter plot surrounded by a red rectangle in the middle panel). Each tensor is not related to other tensors, i.e., the first spatial module is related only to the first temporal module and trial component. (Lower-right) The proportion of the variance in reconstructed data via R tensors to the variance in the original data. The proportion is determined by the uncentered coefficient of determination. The solid black line and black shaded area indicate the mean and standard deviation of the proportion across eight subjects. In major parts of this study, we chose R based on the criteria to explain 80% of the variance of the original data in each subject.

The current study discloses how muscle activities are related to motion effort while focusing on spatial and temporal modules. We examine three factors, spatial modules, temporal modules, and effort dependence. A standard method used to extract spatiotemporal muscle modules is nonnegative matrix factorization (NNMF)^21^. Because NNMF enables us to analyze matrices with two factors (i.e., row and column), it is suitable for two-factor analysis. For (more than) three-factor analysis, tensor decomposition rather than NNMF can be more effective^22^ (Fig. 1). While sorting an array of matrices along the third dimension (K in the left panel in Fig. 1), tensor decomposition enables us to analyze three factors (i.e., the column [S], row [T], and number of slices in the third dimension [K]). Previous studies have shown the effectiveness of tensor decomposition and its variants in discussing task-dependent effects on spatiotemporal muscle modules^23–25^. We thus expect to discuss how spatiotemporal modules show effort-dependent effects by using tensor decomposition for each subject. After extracting the effort dependence of the spatiotemporal modules in each subject, we examined how motion effort affected common and unique EMG features.

## Results

**Figure 2:**
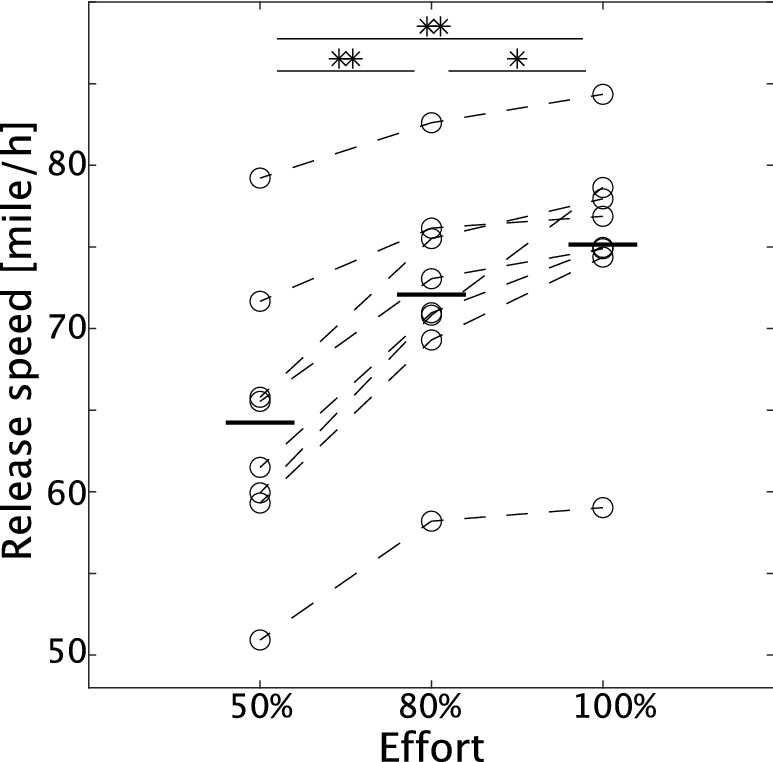
Ball-release speed in each level of effort. The horizontal solid black lines indicate the release speed averaged across all the trials and subjects in each condition (N = 8). Each circle represents the release speed averaged across all the trials in each subject. Each dotted black line indicates ball-release speed in each subject and effort. Single and double asterisks show statistically significant differences with *p* < 0.05 and *p* < 0.01 in Tukey’s comparison test.

Eight pitchers performed natural three-dimensional pitching with wind-up, cocking, acceleration, release, and follow-through phases (i.e., skilled pitching). Our experimental setup was close to a real baseball environment, i.e., 18.4 m from the mound to the home plate, with a catcher but without an opposing batter. The pitchers executed skilled pitching with either 50%, 80%, or 100% effort for ten trials for each level of effort. Among these conditions, there were significant differences in ball-release speed (Fig. 2, *p* = 0.00020 between 50 and 80% effort, *p* = 0.00092 between 50 and 100% effort, and *p* = 0.022 between 80 and 100% effort [Tukey’s comparison test]). In each subject, there were main effects of effort upon ball speed (F(2,18) > 53.6 and *p* < 2.64 × 10−^8^ via one-way ANOVA, with ball speed as a dependent variable and effort as an independent variable in each subject [Fig. S1]). In all the subjects, there was a significant difference in ball speed between 50 and 80% effort (*p* < 2.28 × 10−^4^ via Tukey’s comparison test). Although there was a significant difference in ball speed between 80 and 100% effort in 6 out of 8 subjects (*p* < 0.00762 via Tukey’s comparison test), there was no significant difference in the same comparison in the remaining two subjects (*p* > 0.106 via Tukey’s comparison test). These results indicated the success of performing skilled pitching while classifying motion into 50% and 80% effort and a slight difficulty in performing skilled pitching while classifying motion into 80% and 100% effort.

We focused on the effort-dependent effects on EMG activities from muscles in the trunk, upper body, and both arms (Table 1). The current study analyzed the EMG activities for 750 ms or resampled 150 time frames including ball-release timing at the 150th time frame. These timings include the stride, cocking, acceleration, and release phases in skilled pitching^15^. In particular, the current study concentrated on the spatial module (i.e., groups of muscles showing correlated activity), temporal module (i.e., time-varying recruitment pattern of the spatial module), and trial component (i.e., how each spatiotemporal module is recruited in each level of effort). To evaluate these three factors, we applied CANDECOMP/PARAFAC (CP) decomposition to the EMG activity data for each subject. Because EMG data were nonnegative, we utilized CP decomposition with nonnegative constraints for all values.

**Table 1:** We measured and analyzed the muscles summarized in this table on the right and left sides of the pitchers.

FCU: Flexor carpi ulnaris	ECR: Extensor carpi radialis longus	BB: Biceps brachii
TB: Triceps brachii	DL: Deltoid middle strand	PM: Pectoralis major
TU: Upper part of the trapezius	TU: Upper part of the trapezius	EO: External oblique

**Figure 3:**
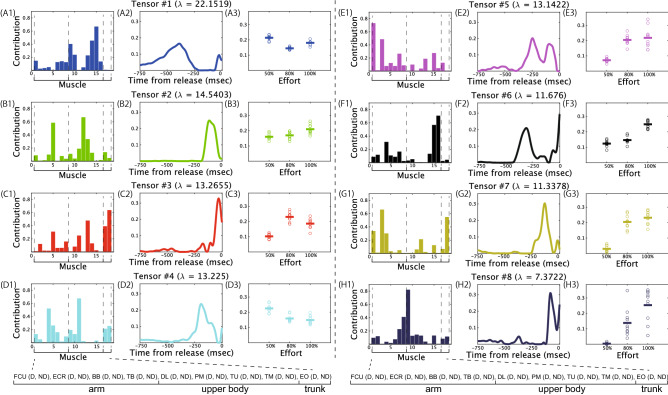
Spatial modules, temporal modules, and trial components extracted via CP decomposition with nonnegative constraints in a typical subject. $\uplambda$ indicates the contribution of each tensor in reconstructing the original data (detailed descriptions were provided in the Materials and methods section). Each color indicates the associated combinations of the spatial module, temporal module, and trial component. For example, the blue-colored spatial module in panel A1 is associated with the blue-colored temporal module in panel A2 and the blue-colored trial component in panel A3. (A1–H1): Extracted spatial modules. Horizontal and vertical axes indicate the muscle number and the recruitment of each muscle in the spatial module. Each muscle number corresponds to the name listed at the bottom part of this figure. The definitions for the abbreviated muscle names in the bottom part of this figure are listed in Table 1. The abbreviations D and ND associated with each muscle indicate the dominant and nondominant side. (A2–H2): Extracted temporal modules. Horizontal and vertical axes indicate the time frame number and the recruitment magnitude. (A3–H3): Extracted trial components. Horizontal and vertical axes indicate the effort type and the magnitude of the trial component. The trial component in each trial is shown as a dot. The horizontal solid lines indicate the trial component averaged across trials in each level of effort. Larger values in the component indicate larger recruitment of the associated spatial and temporal modules in that level of effort.

Figure 3 demonstrates the extracted spatial modules, temporal modules, and trial components in a typical subject. The combination of the spatial module, temporal module, and trial component is hereafter referred to as the tensor. The current study determined the number of tensors based on the criteria to explain 80% of the variance of the original data (the lower-right panel in Fig. 1). Of note, the number of extracted modules in the criteria was different in each subject (6–14, mean ± standard deviation was 10.375 ± 2.774). We also discussed the influence of the criteria on our results in a later section and in the supplementary material. In the CP decomposition, the spatial module is associated only with the temporal module and the trial component within each tensor. In other words, the blue-colored spatial module in Fig. 3A1 was associated with the blue-colored temporal module (Fig. 3A2) and the blue-colored trial component (Fig. 3A3) in tensor #1. Differences in colors indicate different groups of the associated spatial module, temporal module, and trial component.

Tensor #1 indicated the spatiotemporal module possibly around the middle of the stride phase while moving the throwing arm backward, based on the increased recruitment pattern in the temporal module approximately 500–350 ms before releasing the ball (Fig. 3A2). We interpreted the peak timing of each temporal module by comparing it to the pitching phases shown in a previous study^15^. In the spatial module, upper body muscles were recruited (middle part of the trapezius (TM) on the dominant side, upper part of the trapezius (TU) on the nondominant side, and deltoid middle strand (DL) on the dominant side, Fig. 3A1). These spatiotemporal modules were recruited across the three types of effort, although the recruitment pattern was slightly larger in 50% effort trials (Fig. 3A3).

Based on the peak timings of the temporal modules, the spatiotemporal module recruited after tensor #1 was tensor #6, although tensor #6 was recruited again around the time that the ball was released (Fig. 3F2). The temporal module showed large activation patterns at approximately 350–250 ms before the ball was released or before the transition from the stride to the arm-cocking phase (Fig. 3F2). In the spatial module, upper body muscles were recruited (TM in both dominant and nondominant sides, Fig. 3F1). These modules were largely recruited in 100% effort trials compared to 50% and 80% effort trials.

Tensors #2, #4, #5, #7, and #8 represented spatiotemporal modules around the arm-cocking phase (i.e., 200–50 ms before release, Fig. 3B,D,E,G,H). In the spatial modules, large recruitment patterns were observable in both the upper body and arm muscles (the biceps brachii (BB) in the dominant side and the pectoralis major (PM) in the nondominant side in tensor #2, the extensor carpi radialis longus (ECR) in the nondominant side and the PM in the dominant side in tensor #4, Fig. 3B1 and 3D1), arm muscles (the flexor carpi ulnaris (FCU) in the dominant side and ECR in the dominant side in tensor #5, Fig. 3E1), both arm and trunk muscles (the FCU in the dominant side, the ECR in the dominant side, and the external oblique (EO) in the nondominant side in tensor #7, Fig. 3G1), or upper body muscles (the DL in the dominant side and the triceps brachii (TB) in the nondominant side in tensor #8, Fig. 3H1). In tensors #2 and #4, the trial components indicated slight effort-dependent effects on the recruitment patterns of the spatiotemporal modules (Fig. 3B3 and D3). In contrast, in tensors #5, #7, and #8, large effort-dependent effects were evident (Fig. 3E3, G3, and H3).

Around release (i.e., around the 750th time frame), the recruitment of the spatiotemporal module in tensor #3 was obvious (Fig. 3C), based on the peak timing of the temporal module (Fig. 3C2). Both the upper body and trunk muscles were related (the TU on the dominant side and the EO on both sides, Fig. 3C1). The trial component denoted larger recruitment in 80% and 100% effort trials than in 50% effort trials.

In summary, CP decomposition enabled us to find several effort-dependent effects on the spatiotemporal modules, such as those observed in tensors #3, #5, #7, and #8 in a typical subject. Thus, CP decomposition allowed us to evaluate the effort-dependent effects on the time-varying and multiple muscle activities in skilled pitching. The results in Fig. 3 allowed us to interpret how the effort-dependent effects were apparent in each phase and muscle activation pattern.

**Figure 4:**
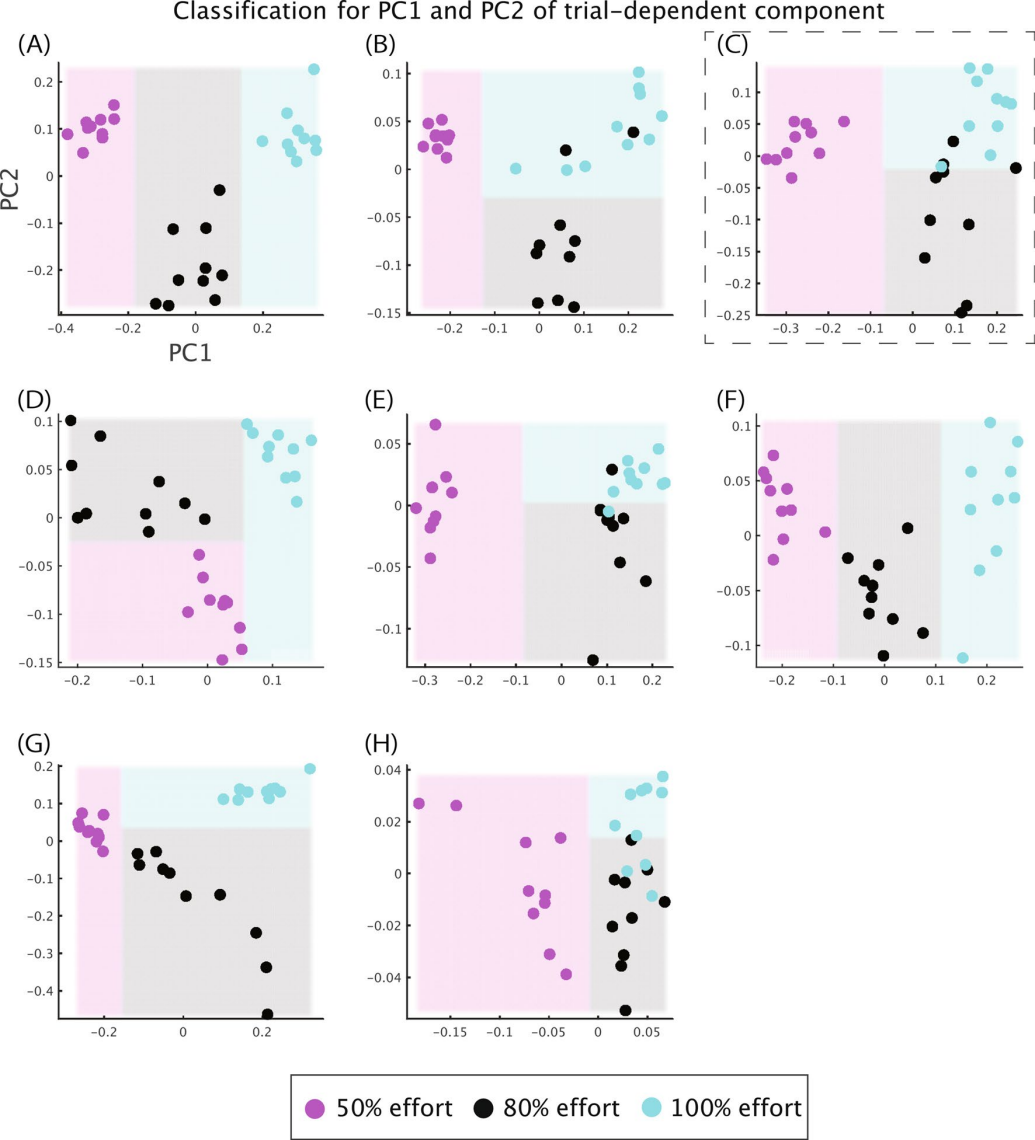
PC1 and PC2 of the trial components in each subject and the decision boundaries estimated by a classification tree algorithm. Horizontal and vertical axes indicate PC1 and PC2. Magenta, black, and cyan dots demonstrate the PC1 and PC2 values in each trial with 50%, 80%, and 100% effort, respectively. Magenta, black, and cyan areas denote the data area to be classified into 50%, 80%, and 100% effort estimated by the classification tree algorithm. Panels (**A**–**H**) denote the PC1 and PC2 values in each subject. Panel (**C**) surrounded by the black dotted rectangle corresponds to the typical subject in Fig. 3.

We then investigated the tendencies across whole subjects to examine how uniform and unique motion features in each individual depended on motion effort. Nevertheless, there was a problem: the extracted spatial modules, temporal modules, trial components, and the number of extracted tensors were different among all the subjects. To overcome this problem, we derived low-dimensional structures inherent in the trial components based on the following guidelines. CP decomposition permitted us to extract spatial modules, temporal modules, and trial components without orthogonal constraints. That is, some overlaps between spatial modules, temporal modules, and trial components were allowed. Due to these nonorthogonal properties, the extracted spatiotemporal modules indicated similar effort-dependent effects (e.g., in tensors #3, #5, #7, and #8). An advantage of this nonorthogonality was that we were able to discuss how each spatiotemporal module was recruited depending on effort in detail. On the other hand, a disadvantage associated with the nonorthogonality of this approach was the larger number of extracted tensors than the number of tensors extracted via the analysis with orthogonal constraints. A smaller number of tensors provides better interpretability. The current study thus extracted useful and lower-dimensional features to discuss effort-dependent effects on time-varying multiple muscle activities across the subjects using a method with orthogonal constraints. In particular, we applied principal component analysis (PCA) to extract the most and second-most prominent dimensions intrinsic to trial components (Fig. 4, see Methods for details). The two dimensions allow us to visualize the results in an interpretable manner. After extracting the two dimensions in all the subjects, we then compared the dimensions among the subjects.

The two essential dimensions of the trial components explained 75–91% of the variance in the original trial components (85.4 ± 5.9% [mean ± standard deviation]). If each trial component uniformly incorporated the original information, the two dimensions would explain 10.8% of the variance. In contrast to this uniform assumption, the extracted two dimensions include enough detail to interpret the original trial components. We then applied a classification tree algorithm to discuss effort-dependent effects in the two dimensions (Fig. 4). One of the most powerful features of the tree algorithm is its interpretability. The classification error was 0–0.2 (0.0917 ± 0.0792 [mean ± standard deviation]), indicating approximately 90% classification accuracy. Because there were three categories (i.e., 50%, 80%, and 100% effort), a random classifier would demonstrate a classification error of 0.67. The two dimensions of the trial components possessed enough details to discuss both the original trial components and the difference among the three levels of effort.

In six out of eight subjects (Fig. 4B–E,G,H), two dimensions were used to classify three types of effort. In five of these six subjects (Fig. 4B,C,E,G,H), the first principal component (PC1) in the trial components was effective in distinguishing between low effort (i.e., 50%) and high effort (i.e., 80% and 100%), and the second principal component (PC2) was efficient in classifying high effort (i.e., 80%) and the highest effort (i.e., 100%). In the remaining subject (Fig. 4D), it was possible to distinguishing between 100% effort and other levels of effort using PC1, and PC2 allowed us to classify 50% and 80% effort. In the remaining two subjects (Fig. 4A,F), one dimension was enough to classify the level of effort. Among seemingly diverse features used to classify effort, it was possible to find consistency. Although PC2 was not utilized in the classification tree in Fig. 4A and 4F, 80% effort trials were associated with a lower value of PC2, and 100% effort trials were associated with a more substantial value of PC2—the features in Fig. 4A,F were thus consistent with the elements in Fig. 4B,C,E,G,H, in which PC2 was effective in classifying 80% and 100% effort. The subject denoted in Fig. 4D shows the exchanged roles of PC1 and PC2 compared to other subjects. If we exchanged the role of PC1 with that of PC2, the exchanged PC2 (i.e., the original PC1) worked to classify between 50% and other efforts (i.e., 80% and 100%), and the exchanged PC1 (i.e., the original PC2) worked to classify 80% and 100% effort. In sum, two dimensions were sufficient to classify three types of effort in all the subjects. One dimension, PC1 in major subjects, could be used to successfully classify the effort into 50% effort and high effort. The other dimension, PC2 in major subjects, showed a tendency to classify the high effort into 80% and 100%.

The current study further investigated the spatial and temporal modules related to classifying three levels of effort. Using the PC1 and PC2 coefficients of the trial components, we calculated the spatiotemporal modules associated with PC1 and PC2 (see Methods for details). The spatiotemporal modules linked with PC1 could be related to classifying low (i.e., 50%) and high effort (i.e., 80% and 100%). Similarly, the spatiotemporal modules related to PC2 could be related to classifying high effort (i.e., 80%) and the highest effort (i.e., 100%). Of note, to compute the average across all the subjects and statistical features, PC1 and PC2 in Fig. 4D were reversed because these roles were reversed compared to their roles in other subjects. Despite the nonnegative constraints in the spatiotemporal modules (Fig. 3), the PC1 and PC2 coefficients included negative values. Thus, the spatiotemporal modules associated with PC1 and PC2 incorporated both positive and negative values. We sorted the sign of spatiotemporal modules associated with PC1 and PC2 without affecting any results (see Methods for details). After sorting the signs, the current study examined the muscles and timings that could be used classify effort.

**Figure 5:**
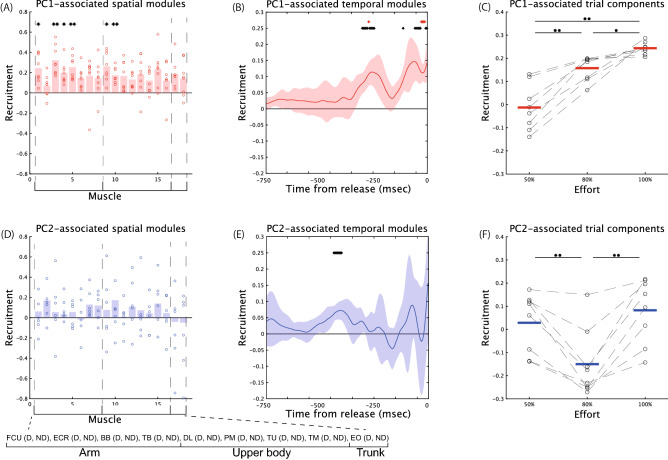
The spatiotemporal modules associated with PC1 and PC2 of the trial components. (**A**–**C**): PC1-associated spatial module, temporal module, and trial component. Double asterisks and single asterisks in panel (**A**) indicate significant differences from 0 with *p* < 0.01 and *p* < 0.05 through a *t*-test with Bonferroni’s correction. Red and black asterisks in panel (**B**) demonstrate significant differences from 0 with *p* < 0.01 and *p* < 0.05 via a *t*-test with Bonferroni’s correction. The muscle type associated with the muscle number in panel (**A**) is listed at the bottom part of this figure. The dotted lines in panel (**C**) indicate the effort-dependent effects on trial components in each subject. Double asterisks above the horizontal solid black line demonstrate a significant difference with *p* < 0.01 (Tukey’s comparison test). A single asterisk indicates a significant difference with *p* < 0.05. (**D**–**F**): PC2-associated spatial module, temporal module, and trial component.

The PC1-related trial components showed a clear separation between 50% and higher effort (*p* < 0.0014, Tukey’s comparison test, Fig. 5C). We then investigated the consistency of the PC1-related spatiotemporal modules across the subjects. The current study performed a *t*-test with Bonferroni’s correction to evaluate the muscles and timings different from 0 consistently across the subjects. If there were consistent recruitments of some muscles across the subjects, the associated p-values would be smaller than some criteria (e.g., 0.05 or 0.01). In the PC1-associated spatial module (Fig. 5A), there were significant recruitments of the FCU in the dominant arm (*p* = 0.0328 [corrected], effect size [es] = 1.72), the ECR in the dominant arm (*p* = 0.0070 [corrected], es = 2.24), the ECR in the nondominant hand (*p* = 0.0266 [corrected], es = 1.79), the BB in the dominant hand (*p* = 0.0097 [corrected], es = 2.12), the DL close to the dominant arm (*p* = 0.0237 [corrected], es = 1.82), and the DL close to the nondominant arm (*p* = 0.0055 [corrected], es = 2.33). In the PC1-related temporal module (Fig. 5B), there were significant recruitments around when the ball was released and 300 ms before the moment of release (*p* < 0.05 [corrected], es > 2.35) or at the transition from stride to arm-cocking phases. Fig. S6 summarizes the effect size. Due to the common features in both the spatial and temporal modules, we regarded the PC1-related spatiotemporal module as common EMG features across the subjects rather than diverse EMG features.

The PC2-related trial components showed a clear separation between 80 and 100% effort (*p* = 0.00247, Tukey’s comparison test, Figs. 4, 5F). There was also a significant difference between 50 and 80% effort (*p* = 0.00681). Of note, based on the classification tree, PC2 was not necessary to distinguish 50% effort from higher levels of effort. There was no consistent recruitment in each muscle across the subject (*p* > 0.5212 [corrected], es < 0.968 [Fig. 5D]), indicating diverse individual differences to classify effort into 80% and 100% categories. In the PC2-related temporal module, there were significant recruitments 400 ms before the release (*p* < 0.0366 [corrected], es > 2.42 [Fig. 5E]) or in the middle of the stride phase. Because of the minor common and major unique features in the spatiotemporal modules, we regarded the PC2-related spatiotemporal module as diverse EMG features rather than common EMG features.

The common EMG features (Fig. 5A,B) were evident when switching from 50% effort to higher levels of effort (Fig. 5C). In contrast, unique EMG features were obvious when switching from 80 to 100% effort (Fig. 5D–F). By combining CP decomposition and PCA, we revealed that common and diverse EMG features demonstrated different types of effort dependence.

We heretofore examined the effort dependence of the spatiotemporal modules with the criteria explaining 80% of the variance of the original data to determine the number of tensors. The current study next checked the influence of the criteria on our results. PC1 and PC2 in the trial components were useful for classifying effort into three levels from time-varying and multiple EMG activity data, indicating the significance of PC1 and PC2 to discuss effort-dependent effects on EMG features.

**Figure 6:**
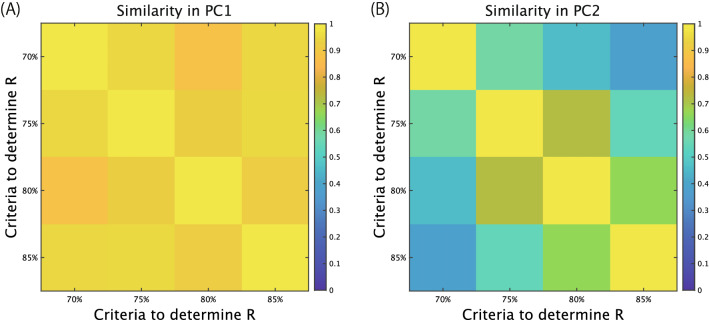
Similarities of PC1 and PC2 in trial components among different criteria to determine the number of tensors. (**A**) Absolute values of Pearson’s correlation of PC1 coefficients. (**B**) Absolute values of Pearson’s correlation of PC2 coefficients.

Thus, we investigated the similarities of PC1 and PC2 in the trial components across different criteria. The PC1 values were highly consistent across the criteria to explain 70%, 75%, 80%, and 85% of the variance in the original data (Fig. 6A, the absolute value of the correlation coefficients averaged across the subjects [abbreviated as absolute correlation hereafter] were larger than 0.8774). In contrast, the PC2 values were more inconsistent than those in PC1 (Fig. 6B, the absolute correlations were larger than 0.3807). In particular, in PC2, there was a large difference between the criteria to explain the lowest (i.e., 70%) and the highest (i.e., 85%) variance (the absolute correlation was 0.3807). Between the criteria regarding 75% and 80% variance (the absolute correlation was 0.7216) or 80% and 85% variance (the absolute correlation was 0.6603), the tendencies in Fig. 4 were not significantly different from the perspective of the absolute correlation. Despite the lower absolute correlations in PC2 than in PC1, the qualitative tendencies were consistent across the four types of criteria (Figs. S2–S5 in Supplementary material). The common EMG features in PC1 across the subjects that classified the effort into 50% and higher levels of effort were invariant across the criteria to explain the variance of the original data. The diverse EMG features in PC2, to classify the effort into 80% and 100% categories, slightly depended on the criteria. Of note, irrespective of this observed dependence on criteria, our results were invariant. The EMG features associated with PC1 were robust against the criteria that determined the number of tensors (Figs. S2-S5), indicating that the commonly observed EMG features (Fig. 5A–C) were also invariant against the criteria. The EMG features associated with PC2 were not robust against the criteria, indicating that the unique EMG features (Fig. 5A–C) were also observable in several thresholds.

## Discussion

The current study extracted the effort-dependent effects on spatiotemporal modules via tensor decomposition (Figs. 1, 3). The extraction allowed us to visualize how the recruitment of each muscle at each time was related to each level of effort or the speed of the fastball (Figs. 2, 3). To examine effort-dependent effects on common and diverse EMG features, we extracted low-dimensional spaces inherent in the trial components that indicated the effort dependence of the spatiotemporal modules. Two dimensions were enough to classify effort into 50%, 80%, and 100% (Fig. 4). One dimension was needed to separate 50% from higher levels of effort, and the other dimension was needed to classify effort as either 80% and 100%. In the dimension used to separate 50% from higher levels of effort, several arm muscles and upper body muscles were consistently recruited approximately 300 ms before the ball was released and around the release timings across subjects (Fig. 5A–C). In the other dimension to separate 80% from 100% effort, there were distinct individual differences in muscle recruitment but slight consistent activations at approximately 400 ms before the moment of release across subjects (Fig. 5D–F).

Although the effort-dependent modulations of spatiotemporal modules were not thoroughly examined, we predicted some possible results. The first possibility was that unique motion features were more evident when a greater effort was exerted in skilled pitching, based on pianist research^14^. The second possibility was that greater effort was associated with more evident common motion features because common features play roles in facilitating faster elbow and wrist rotations^10^. Our results supported the coexistence of the two possible results. In Fig. 5A–C, the dimension to separate 50% from higher effort can consist of common EMG features across subjects rather than unique EMG features. In Fig. 5D–F, the dimension to separate 80% from 100% effort can consist of unique EMG features rather than common EMG features. Our results clarified the common and diverse EMG features and their different effort-dependent effects from time-varying and multiple EMG activity data via CP decomposition.

The common and diverse EMG features were extracted via the combination of CP decomposition and PCA (Figs. 4, 5). A strength of the combination is that it allows us to discuss interpretable low-dimensional features shared across the subjects, which complements the detailed analysis for each subject via CP decomposition. In the dimension to separate 50% effort from higher levels of effort (Fig. 5A–C), there were larger recruitments of the FCU in the dominant arm, the ECR in the dominant and nondominant arms, the BB in the dominant arm, and the DL in the dominant and nondominant sides approximately 300 ms before the ball was released and just before the ball was released in higher levels efforts. In the dominant arms and sides, these larger recruitment patterns when greater effort is exerted correspond to larger angular velocities of the shoulder internal rotation, elbow extension, and wrist flexion when utilizing interaction torques^10, 26^. We can expect similar functional roles of a more considerable involvement of the ECR and the DL in the nondominant arm and side. The extracted common EMG features can thus correspond to the previous findings in kinematics and kinetics. In addition, our analysis sheds light on the effort-dependent effects on pitching features. Furthermore, in addition to thoroughly investigating functional roles on the pitcher’s dominant side, our results indicated significant functional roles on the nondominant side in skilled pitching.

The diverse features extracted via CP decomposition and PCA were essential to separate 80% effort from 100% effort (Fig. 5D–F). Although we found a few common features only in the temporal module approximately 400 ms before the ball was released, there was no consistent tendency in the spatial module across subjects. A possible origin of the individual difference is the difficulty of separating 80% and 100% effort in skilled pitching. In the comparison of release velocity (Fig. 2), there was less of a difference between 80 and 100% effort than between 50% effort and higher levels of effort. In the result of the classification tree (Fig. 4), it was feasible to separate 50% effort from higher levels of effort with perfect accuracy (i.e., the separation of magenta dots from other dots). In contrast, classification accuracy was lower in separating 80% and 100% effort (the separation between black and cyan dots). Another possible origin of the individual difference is a diverse way of increasing effort or ball velocity. Some subjects increased effort and ball velocity while modulating the recruitment of PC1 and PC2 (Fig. 4A,F). Other subjects increased effort and ball velocity while modulating the recruitment of only PC2 (Fig. 4B,C,E,G,H). These different manners to increase effort and ball velocity added to the complexity of detecting common features.

When analyzing a typical subject (Fig. 3), each temporal module showed a different time peak. By focusing only on the muscles whose recruitment pattern in the associated spatial module was large on the dominant side, the following sequential activation patterns were observed: TM (in tensors #1 and 6 approximately 400 ms before the ball was released), FCU and ECR (in tensor #5 approximately 250 ms before the ball was released), PM (in tensor #4 approximately 200 ms before the ball was released), BB and ECR (in tensors #2 and #7, respectively, approximately 120 ms before the ball was released), FCU, ECR, TB, and DL (in tensors #5 and #8 approximately 70 ms before the ball was released), EO and TU (in tensor #3 approximately 15 ms before the ball was released), and TM (in tensor #6 around the time the ball was released). Effort-dependent effects were observable in tensors #3, #5, #7, and #8. For example, in tensor #7, the ECR was recruited in the arm-cocking phase depending on effort. This effort-dependent recruitment of the wrist extensor muscle may reflect a more significant wrist flection velocity in proportion to throwing speed^10, 26^ after a more considerable wrist extension. In tensor #7, the EO in the nondominant side also demonstrated more substantial involvement, possibly starting to yield more significant angular velocity in shoulder internal rotation. For another example, in tensor #3, the recruitment of EO in the dominant and nondominant sides just before the ball was released may reflect the use of a more significant rotation to facilitate larger angular velocity in distal parts^10, 26^. Additionally, as another example, in tensor #6, the recruitment of the TM in the dominant and nondominant sides just around the time of release were evident. In addition, this recruitment was larger in cases of 100% effort than in other levels of efforts. These more considerable TM involvements on both sides may also indicate a larger angular velocity in shoulder internal rotation. CP decomposition enables us to discuss effort-dependent effects on these aspects while considering cooperatively activated muscles. In particular, we observed the cooperative activity of muscles on the dominant side and nondominant side through effort-dependent effects on spatiotemporal modules. If we wanted to focus on the sequential activation patterns within a specific level of effort, such as in previous works^8, 15^, we should focus on trials with one level of effort. CP decomposition allows us to discuss the features inherent in the peak timings and onset time while considering spatial modules. Furthermore, if we wanted to focus on the variability of the speed of the fastball within a specific level of effort, as in previous studies^6, 7^, we should focus on one level of effort.

Although the 80% criterion is a standard criterion threshold for extracting spatiotemporal modules in EMG data, determining the number of modules or tensors is an ongoing question in PCA, NNMF, and CP decomposition (see^27^). Thus, we compared the robustness of our results against the criteria to determine the number of modules. The dimension to separate 50% from higher effort was robustly invariant against the criteria (Fig. 6A). The difference between 50% and higher efforts was evident not only in the ball-release velocity (Fig. 2) but also in the trial components (Figs. 3, 4, 5). Compared to this dimension, the dimension to separate 80% effort from 100% effort was influenced by the criteria (Fig. 6B). A possible reason is that a difference between 80 and 100% effort was less evident than the difference between 50% effort and higher levels of effort in the ball-release speed (Fig. 2). Another reason is that the effort-dependent effects of the spatiotemporal modules emerged in tensors whose contributions to explaining the original data were less, such as in tensors #6, #7, and #8. If we changed the criteria, the trial components with effort-dependent effects changed to some degree, resulting in a fluctuating representation. Of note, although the representation in the dimension used to separate 80% from 100% effort was influenced more strongly by the aforementioned criteria than was the dimension used to separate 50% from higher efforts, the dimension used to separate 80% from 100% effort showed a degree of consistency with a correlation coefficient larger than 0.66 among the criteria used to explain 75%, 80%, and 85% of the variance of the original data. Additionally, the dimension to separate 80% effort from 100% effort showed qualitative consistencies (Figs. S2–S5).

A limitation of tensor decomposition is that the number of the extracted spatiotemporal modules is larger than that for PCA or NNMF (but tensor decomposition has a lower number of parameters compared to these methods, see Methods for details). The larger number of modules often causes complicated interpretations of the modules—the current study utilized PCA to overcome this disadvantage (Figs. 4, 5). PCA provided a clear interpretation by reducing the dimensionality, but nonnegative constraints were removed instead. Another limitation of tensor decomposition is its difficulty in addressing task-relevant and task-irrelevant components except for some special cases. The spatiotemporal module framework is a way to highlight how to manage a large number of DoF in the human body. Another perspective on the redundancy problem is the decomposition of motion data into task-relevant and task-irrelevant components^28, 29^ and the suppression of motor variability, especially in task-relevant motion components^29^. To discuss task-relevant and task-irrelevant features, different types of data-driven methods^30–32^ are preferable rather than tensor decomposition. Because the current study focused on the effort-dependent effects on multiple and time-varying muscle activities, we relied on tensor decomposition. Modifications to tensor decomposition are also possible while allowing the association of one spatial module with more than one temporal module by different weight values^22, 24, 25^ (i.e., Tucker decomposition and its variants). In the cases of extracting smoother temporal modules, it is also possible to define smooth constraints in temporal aspects^33–35^. Depending on the research purpose, we should use appropriate data analysis methods.

In conclusion, we clarified the following two features: (1) how spatial and temporal modules in baseball pitching motion were common and individually different, and (2) the effort-dependent modulations of the common and unique modules. Tensor decomposition was crucial for clarifying these features by analyzing time-varying EMG data in multiple muscles at three levels of motion effort simultaneously.

## Materials and methods

### Participants

Nine subjects participated in our experiment (nine males, two left-handed and seven right-handed; age 19–49 years; height 167–180 cm; bodyweight 70–82 kg; and baseball pitching experience for 5–38 years). They abstained from physical activities 24 hours before participating in the experiments. Of note, we did not find an influence of age on our results. For example, Fig. 4B,G,H denote the essential dimensions of EMG modulations for subjects aged 21, 49, and 25 years old, respectively. In these subjects, one dimension was related to classifying motion into 50% and 80% effort, and the other dimension was related to classifying motion into 80% and 100% effort. In contrast, one dimension was essential for separating three levels of motion effort in 19- and 24-year-old subjects, denoted in Fig. 4A,C, respectively. In summary, the age-dependent difference was not as significant as the individual difference.

Our inclusion criteria were that all of the subjects were trained as baseball pitchers for more than five years, and they participated in university or Japanese professional baseball leagues as pitchers. We excluded one out of the nine subjects because the measured EMG data in more than ten muscles were larger than the mean + 3 $\times$ standard deviation in more than ten trials. In total, we focused on eight subjects. Three of them were baseball pitchers in a Japanese university baseball league. Two subjects were retired pitchers that were in a Japanese university baseball league. The remaining three subjects were retired baseball pitchers that were in the Japanese professional baseball league. The experimental procedures were approved by the Ethical Committee of the Graduate School of Arts and Sciences of the University of Tokyo (the approval number was 366–2) and were performed in accordance with the relevant guidelines and regulations. All the participants were informed of the experimental procedures in accordance with the Declaration of Helsinki, and all participants provided written informed consent before the start of the experiments.

### Apparatus and procedure

After an appropriate warmup session, the subjects were instructed to throw a baseball (diameter: 7.2 cm, weight: 145 g) thirty times from a pitching mound toward the low outside corner indicated by both a home plate and a catcher. The experimental environment was almost the same as a real baseball environment, such as having a distance of 18.4 m from the mound to the home plate, but without an actual batter present. When a subject was left-handed, the lower outside corner meant lower and rightward direction from the subject's viewpoint. Similarly, when a subject was right-handed, the lower outer corner meant a lower and leftward location from the subject's viewpoint. The outside corners were based on the assumption that a left (right)-handed pitcher competed with a left (right)-handed batter. We focused on the lower outer corner because it is the furthest location from an opposing batter within the strike zone.

The thirty throws consisted of ten throws with 50% subjective effort, ten throws with 80% subjective effort, and ten throws with 100% subjective effort. We instructed the subjects to throw a baseball with 50% effort during the first ten trials, 80% effort during the next ten trials, and 100% effort during the last ten trials. Because we did not provide any other details, each subject determined the motion effort by themselves. These pitching trials were interleaved with appropriate rest periods depending on the fatigue in each trial and in each subject. The rest periods were approximately 30 s and not more than one minute. The ball-release velocity was measured via Trackman Baseball.

We measured EMG activities from 18 muscles in the trunk, upper body, and both arms with the Trigno Wireless EMG System (Trigno Wireless EMG System; Delsys, Boston, MA, USA) at a 2000 Hz sampling rate. Table 1 summarizes the measured muscles. The electrodes were carefully located to minimize crosstalk from adjacent muscles. To avoid the crosstalk, the inter-electrode distance was set to be 10 mm.

### Preprocessing

After the offset value of EMG data in each muscle was subtracted, the EMG data were digitally full-wave rectified. Following the full-wave rectification, the EMG signal was low-pass filtered with 10 Hz using a built-in IIR filter in MATLAB 2019b. We set the steepness parameter to be 0.99, indicating that the transition width of the filter corresponded to 1% of the difference between the Nyquist frequency and passband frequency. The low-pass-filtered EMG data were resampled at a 200-Hz sampling rate and normalized such that the minimum and maximum values were 0 and 1, respectively. We focused on the 750 ms before the ball was released, i.e., 150 time frames in the resampled EMG. Ball-release timings were detected visually based on the videos measured via a high-speed camera.

### Tensor decomposition

The current study focused on CP decomposition^22^. Let us assume the size of tensor data ${X}_{i,j,k}$ as $S\times T\times K$, where $S$ denotes the number of muscles analyzed, $T$ indicates the number of time frames, and $K$ indicates the number of trials ($i=1,....,S,j=1,...,T,k=1,...,K$). In the framework, tensor data ${X}_{i,j,k}$ is decomposed as

1 $$X_{i,j,k} = \mathop {\mathop \sum \limits_{r = 1} }\limits^{R} s_{i,r} t_{j,r} u_{k,r} ,$$

where $R$ is the number of modules to be determined; ${s}_{i,r}$ is the $i$th element of the $r$th module; ${t}_{j,r}$ is the $j$th element of the $r$th module; and ${u}_{k,r}$ is the $k$th element of the $r$th module. In other forms, the $k$th slice of tensor data is approximated as

2 $$X_{:,:,k} = \mathop {\mathop \sum \limits_{r = 1} }\limits^{R} {\varvec{s}}_{r}^{T} {\varvec{t}}_{r} u_{k,r} ,$$

where ${{\varvec{s}}}_{r}=({s}_{1,r},{s}_{2,r},...{s}_{S,r})$ is the $r$ spatial module (or muscle synergy), ${{\varvec{t}}}_{r}=({s}_{1,r},{s}_{2,r},...{s}_{T,r})$ is the $r$ temporal module, and ${{\varvec{s}}}_{r}^{T}$ is the transpose of ${{\varvec{s}}}_{r}$. CP decomposition thus enables us to estimate how spatiotemporal modules are common or uncommon among all trials through the $r$th trial component ${{\varvec{u}}}_{r}$. If the $r$th spatiotemporal modules were common across all the trials, all the ${u}_{k,r}$ components would have the same value in $k=1,...,K$. If the $r$th spatiotemporal modules were recruited only in the first trials, ${u}_{1,r}$ would have a nonzero value and ${u}_{2,r},...,{u}_{K,r}=0$.

We calculated the spatiotemporal modules and trial components by minimizing the following squared error with nonnegative constraints:

3 $$E = \frac{1}{2}\mathop {\mathop \sum \limits_{i = 1} }\limits^{S} \mathop {\mathop \sum \limits_{j = 1} }\limits^{T} \mathop {\mathop \sum \limits_{k = 1} }\limits^{K} \left( {X_{i,j,k} - \mathop {\mathop \sum \limits_{r = 1} }\limits^{R} \lambda_{r} s_{i,r} t_{j,r} u_{k,r} } \right)^{2} ,$$

where ${\lambda }_{r} \ge 0$ is the scaling factor to let ${{\varvec{s}}}_{r}{{\varvec{s}}}_{r}^{T}=1$, ${{\varvec{t}}}_{r}{{\varvec{t}}}_{r}^{T}=1$, and ${{\varvec{u}}}_{r}{{\varvec{u}}}_{r}^{T}=1$. Due to the nonnegative constraints, ${s}_{i,r}\ge 0$, ${t}_{j,r}\ge 0$, and ${u}_{k,r}\ge 0$. The current study utilized the tensor toolbox for MATLAB and the “cp_nmu” function^36^.

We focused on the criteria to explain 80% of the variance inherent in the original data. The explained variance was calculated as the uncentered coefficient of determination. This 80% criterion seems lower than previous studies with NNMF due to the number of parameters. In our study, $S=18$ (i.e., the number of muscles), $T=150$ (i.e., the number of time frames), and $K=30$ (i.e., the number of trials). If we extracted common spatial modules among all the trials by using NNMF, the number of parameters would equal $R\times S+R\times (T\times K)=\mathrm{36,144}$ when $R=8$. If we extracted spatial and temporal modules in each trial by using NNMF, the number of parameters would equal $(R\times S+R\times T)\times K=\mathrm{40,320}$ when $R=8$. In contrast, in CP decomposition, the number of parameters equaled $R\times S+R\times T+R\times K=\mathrm{1,584}$. Furthermore, larger amounts of criteria can reconstruction the noise that is inherent in data^22^. While considering the influence of the number of parameters and the goal of avoiding noisy reconstruction, we chose the criteria to explain 80% of the original variance.

### Principal component analysis (PCA)

Because CP decomposition does not require any orthogonal constraints, the obtained modules and trial components show (at-a-glance) similarities to each other. To extract more interpretable differences among 50%, 80%, and 100% effort, the current study applied PCA to trial components.

The PCA allowed us to extract low-dimensional components to explain the variance of the original data. The PCA to the trial components provided the following PC1 ${{\varvec{v}}}_{1}$ and PC2 ${{\varvec{v}}}_{2}$:

4 $${\varvec{v}}_{1} = \mathop {\mathop \sum \limits_{r = 1} }\limits^{R} v_{1r} {\varvec{u}}_{r} ,$$

and

5 $${\varvec{v}}_{2} = \mathop {\mathop \sum \limits_{r = 1} }\limits^{R} v_{2r} {\varvec{u}}_{r},$$

where ${v}_{1r}$ and ${v}_{2r}$ represent the coefficients used to calculate ${{\varvec{v}}}_{1}$ and ${{\varvec{v}}}_{2}$, respectively. Trial components projected onto ${{\varvec{v}}}_{1}$ and ${{\varvec{v}}}_{2}$ explained the largest and the second largest portion of the variance in the covariance matrix of the trial components, respectively. That is, blending trial components via ${v}_{1r}$ and ${v}_{2r}$ provided the dimensions to explain differences among the trials with 50%, 80%, and 100% effort. We thus applied the most and the second-most informative coefficients ${v}_{1r}$ and ${v}_{2r}$ to spatiotemporal modules. The PC1-associated spatiotemporal modules were calculated as $\frac{1}{\left|{\sum }_{r=1}^{R}{v}_{1r}{{\varvec{s}}}_{r}\right|}{\sum }_{r=1}^{R}{v}_{1r}{{\varvec{s}}}_{r}$ and $\frac{1}{\left|{\sum }_{r=1}^{R}{v}_{1r}{{\varvec{t}}}_{r}\right|}{\sum }_{r=1}^{R}{v}_{1r}{{\varvec{t}}}_{r}$, whose norms were normalized to 1. We calculated the PC2-associated spatiotemporal modules in a similar manner. Of note, ${v}_{1r}$ and ${v}_{2r}$ were estimated without nonnegative constraints to discuss the most and the second most informative dimensions. Some of the terms in PC1- and PC2- associated spatiotemporal modules thus took negative values.

For visualization in Fig. 4, we sorted the signs of the PC1 values so that the projected trial components associated with 100% effort were larger than those associated with 50% effort trials in PC1. We also sorted the signs of the PC2 values so that the projected trial components associated with 100% effort were larger than those associated with 80% effort trials in PC2. Of note, these sorting procedures were for mere visualization: the results were invariant independent of these procedures. In detail, PCA enables us to decompose the original data as the multiplication of each PC component as $\sum_{i}{\lambda }_{i}{{\varvec{v}}}_{i}{{\varvec{v}}}_{i}^{T}$. Multiplying each ${{\varvec{v}}}_{i}$ by -1 does not change any of the PCA results.

To compare the PC1- and PC2-associated spatiotemporal modules among the subjects, the signs of the modules should be sorted. After sorting, averaging the modules across all the subjects and statistical comparisons provided meaningful information. We sorted the signs as follows. We checked whether both the spatial module and temporal module were different from 0 based on the *t*-test with *p* < 0.05. If both modules were significantly different 0 and the averages of both modules were negative, we multiplied both modules by -1. Because the original data were decomposed into the products of the spatial module, temporal module, and trial component, there was no change if two of them were multiplied by -1.

### Classification tree

After determining the optimal and minimum leaf size in each subject, we performed the classification tree algorithm. The algorithm enables us to determine the decision boundary in each dimension and calculate the 30% hold-out validation error.

### Statistical analysis

To compare ball-release speed in each level of effort, we performed Tukey’s comparison test. We also utilized Tukey’s comparison test to analyze the classification-relevant trial components in Fig. 5C,F. In analyzing classification-relevant spatial and temporal modules (Fig. 5A,B,D,E), the current study utilized a *t*-test with Bonferroni’s correction. All statistical tests were performed using MATLAB 2019b.

To discuss the recruitment of each component in the PC1- and PC2-associated spatiotemporal modules (Fig. 5), we performed a *t*-test with Bonferroni’s correction. The effect size was determined using Cohen’s d.

## Supplementary information

Supplementary information.

## Data Availability

The datasets analyzed in the current study are available from the corresponding author upon reasonable request. The MATLAB code for CP decomposition is available on the website of the corresponding author.
