# Supplementary material for: Effort-dependent effects on uniform and diverse muscle activity features in skilled pitching
Source: Sci Rep. 2021 Apr 15;11:8211. doi: 10.1038/s41598-021-87614-z (PMC8050268; doi:10.1038/s41598-021-87614-z)
Supplement: Supplementary file 1 — Supplementary information. [file 41598_2021_87614_MOESM1_ESM.pdf]

Supplementary information for "Effort-dependent effects of uniform and diverse muscle activity features in skilled pitching**".**

Tsubasa Hashimoto[1], Ken Takiyama[1*], Takeshi Miki[2], Hirofumi, Kobayashi[2], Daiki Nasu[3], Tetsuya Ijiri[2], Masumi Kuwata[2], Makio Kashino[3], Kimitaka Nakazawa[2]

1 … Tokyo University of Agriculture and Technology, Department of Electrical Engineering and Computer Science, 2-24-16, Nakacho, Koganei, Tokyo, Japan
2 …The University of Tokyo, Department of Life Sciences, Graduate School of Arts and Sciences, Tokyo, Japan
3 … NTT Communication Science Laboratories, Nippon Telegraph and Telephone Corporation, Kanagawa, Japan
*e-mail … t.j.ken.takiyama@gmail.com

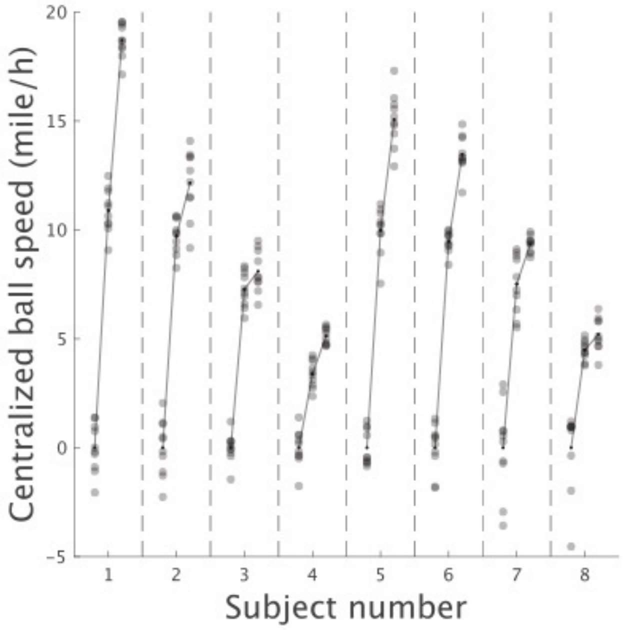

Fig. S1: Ball-release speed in each subject and for each effort. Horizontal and vertical axes indicate the subject number and centralized ball-release speed, respectively. Because the average speed differs in each individual, we subtracted the average speed in 50%-effort trials from the ball-release speed for each subject. For each subject number, each dot indicates the centralized speed. The dots are plotted at three different horizontal locations for each subject. The horizontal locations indicate the trials with 50%, 80%, or 100% motion effort from left to right.

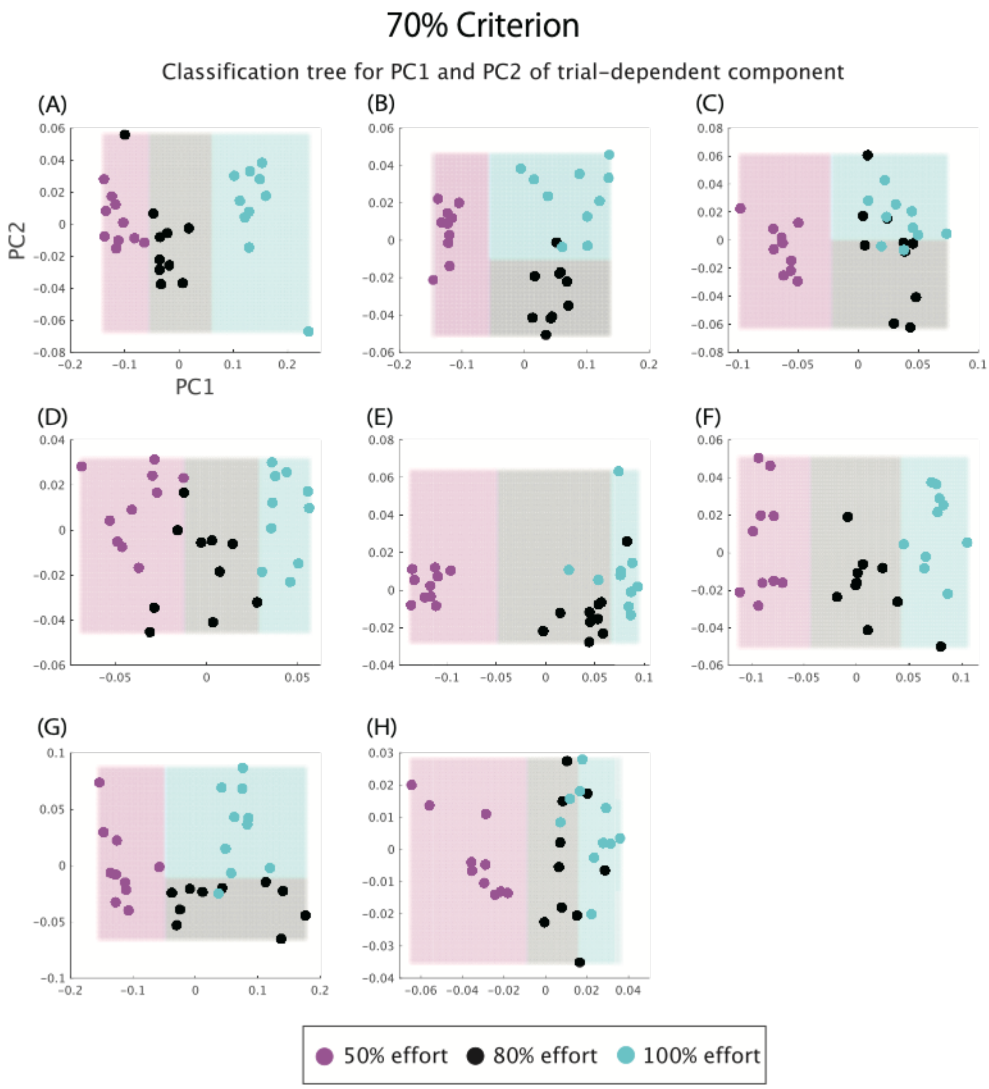

Fig. S2: PC1 and PC2 in the trial components, with the criteria explaining 70% of the variance of the original data. Magenta, black, and cyan dots demonstrate the PC1 and PC2 values in each trial with 50%, 80%, and 100% effort, respectively. Magenta, black, and cyan areas denote the data area classified as 50%, 80%, and 100% effort, estimated by a classification tree algorithm.

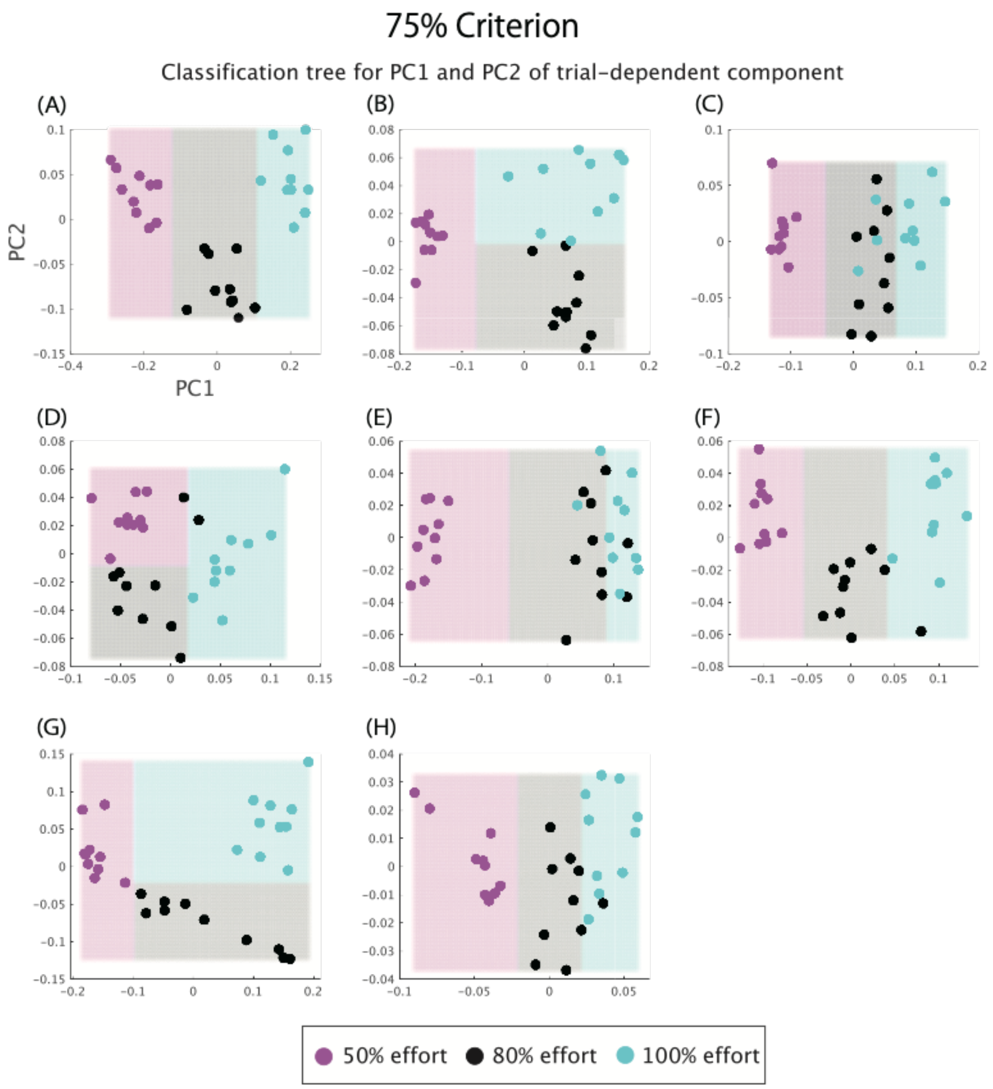

Fig. S3: PC1 and PC2 in the trial components, with the criteria explaining 75% of the variance of the original data.

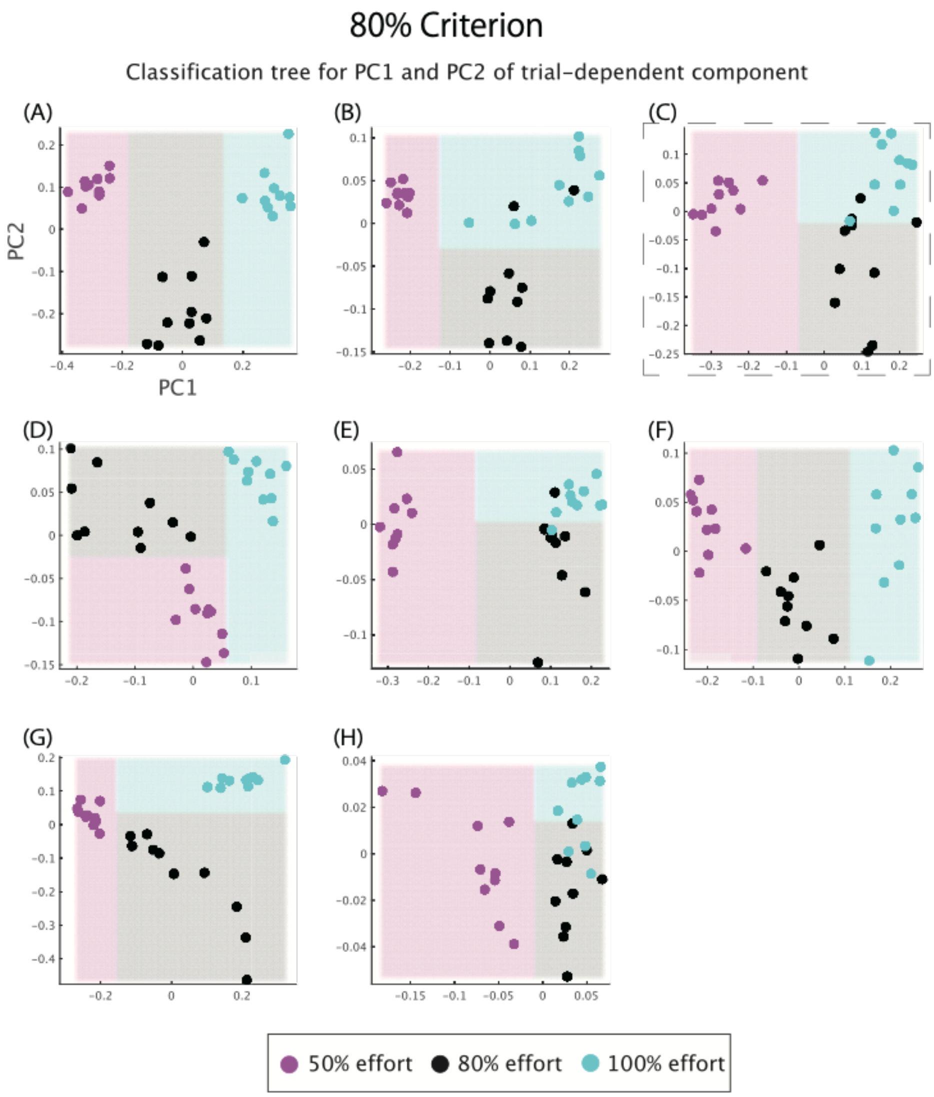

Fig. S4: PC1 and PC2 in the trial components, with the criteria explaining 80% of the variance of the original data. This figure is the same as Fig. 4 in the main text.

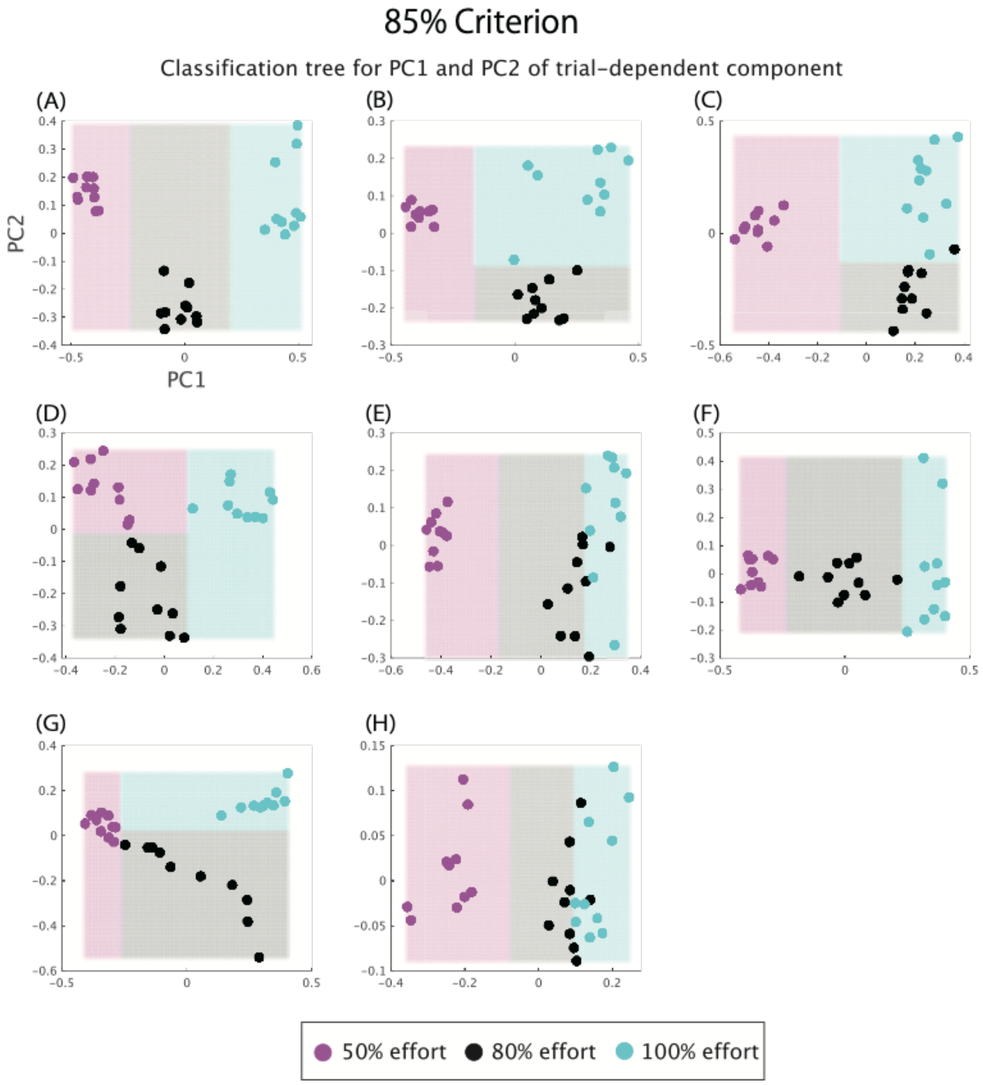

Fig. S5: PC1 and PC2 in the trial components, with the criteria explaining 85% of the variance of the original data.

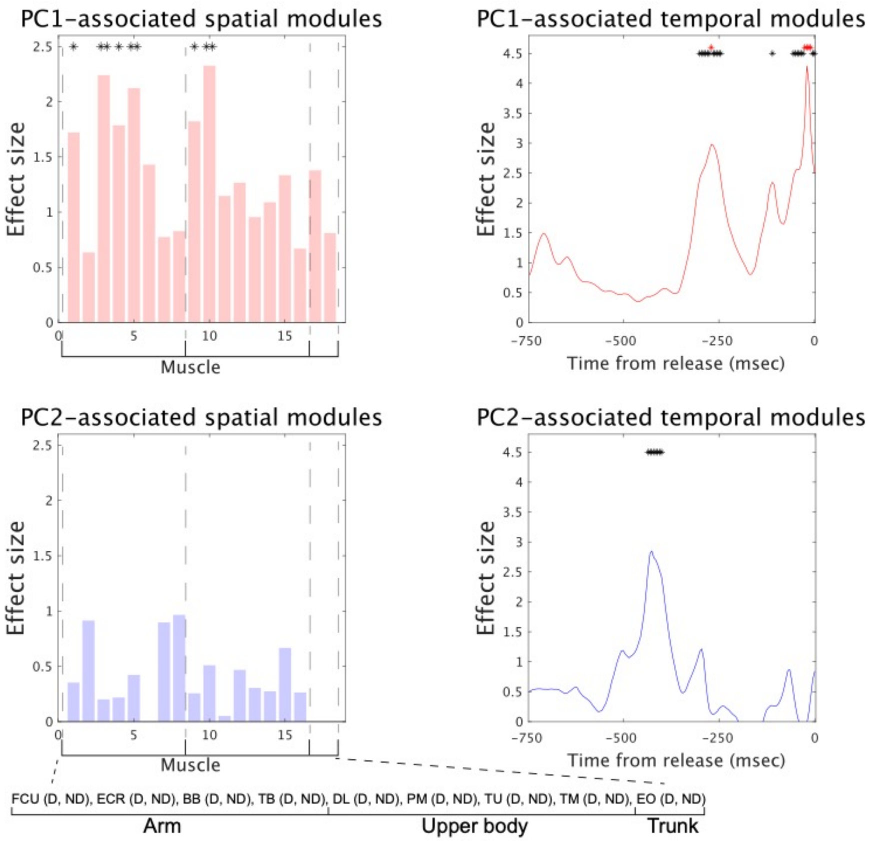

Fig. S6: Effect size of the data shown in Fig. 5. The effect size was calculated based on Cohen's d. Single black asterisks, single red asterisks, and double black asterisks correspond to the same asterisks in Fig. 5.
